# Degeneration of spinal motor neurons by chronic AMPA-induced excitotoxicity in vivo and protection by energy substrates

**DOI:** 10.1186/s40478-015-0205-3

**Published:** 2015-05-14

**Authors:** Citlalli Netzahualcoyotzi, Ricardo Tapia

**Affiliations:** División de Neurociencias, Instituto de Fisiología Celular, Universidad Nacional Autónoma de México, AP 70-253, 04510 México DF, México

**Keywords:** AMPA, Amyotrophic lateral sclerosis, Antioxidants, Energy substrates, Excitotoxicity, Motor neurons, Neurodegeneration, Spinal cord

## Abstract

**Introduction:**

Several data suggest that excitotoxicity due to excessive glutamatergic neurotransmission may be an important factor in the mechanisms of motor neuron (MN) death occurring in amyotrophic lateral sclerosis (ALS). We have previously shown that the overactivation of the Ca^2+^-permeable α-amino-3-hydroxy-5-methyl-4-isoxazole propionate (AMPA) glutamate receptor type, through the continuous infusion of AMPA in the lumbar spinal cord of adult rats during several days, results in progressive rear limb paralysis and bilateral MN degeneration. Because it has been shown that energy failure and oxidative stress are involved in MN degeneration, in both ALS and experimental models of spinal MN degeneration, including excitotoxicity, in this work we tested the protective effect of the energy substrates pyruvate and β-hydroxybutyrate (βHB) and the antioxidants glutathione ethyl ester (GEE) and ascorbate in this chronic AMPA-induced neurodegeneration.

**Results:**

AMPA infusion induced remarkable progressive motor deficits, assessed by two motor tasks, that by day seven reach bilateral rear limb paralysis. These effects correlate with the death of >80% of lumbar spinal MNs in the infused and the neighbor spinal cord segments, as well as with notable astrogliosis in the ventral horns, detected by glial fibrillary acidic protein immunohistochemistry. Co-infusion with pyruvate or βHB notably prevented the motor deficits and paralysis, decreased MN loss to <25% and completely prevented the induction of astrogliosis. In contrast, the antioxidants tested were ineffective regarding all parameters analyzed.

**Conclusions:**

Chronic progressive excitotoxicity due to AMPA receptors overactivation results in MN death and astrogliosis, with consequent motor deficits and paralysis. Because of the notable protection against these effects exerted by pyruvate and βHB, which are well established mitochondrial energy substrates, we conclude that deficits in mitochondrial energy metabolism are an important factor in the mechanisms of this slowly developed excitotoxic MN death, while the lack of protective effect of the antioxidants indicates that oxidative stress seems to be less significant factor. Because excitotoxicity may be involved in MN degeneration in ALS, these findings suggest possible preventive or therapeutic strategies for the disease.

## Introduction

Amyotrophic lateral sclerosis (ALS) is a lethal neurodegenerative disorder characterized by the death of the upper motor neurons (MNs) in the motor cortex and the lower ones in the brain stem and the spinal cord. ALS patients develop a progressive muscle paralysis and there is still no effective treatment. About 10% of ALS cases involve genetic alterations and are therefore termed familial ALS, but the etiology of the remainder 90%, sporadic ALS, is still largely unknown. Among the many factors that have been involved in the mechanisms of MN degeneration are glutamate receptor-mediated excitotoxicity, oxidative stress, mitochondrial dysfunction, neuroinflammation, immune-mediated processes, protein aggregation and axonal transport alterations [1,2], but there is still no satisfactory explanation of why such factors damage predominantly MNs.

Mitochondrial oxidative metabolism seems to be involved in ALS pathogenesis, since morphological and functional alterations in mitochondria of spinal MNs and skeletal muscle of ALS patients have been described [3-6], as well as mitochondrial swelling and vacuolization in the spinal cord of transgenic mutant superoxide dismutase 1 (SOD1) ALS mice [7,8]. In addition, isolated mitochondria from the spinal cord of these mutant SOD1 mice showed decreased Ca^2+^ buffering capacity [8], and dysfunction of oxidative phosphorylation and consequently decreased ATP synthesis [9,10].

The excitotoxic process involves the entrance of Ca^2+^ through glutamate receptors, and the resulting disruption of Ca^2+^ homeostasis activates lytic enzymes, induces the overproduction of reactive oxygen species and leads to mitochondrial dysfunction and energy failure [2,11-13]. Work from our laboratory has shown that such excitotoxic mechanism, mediated mainly by overactivation of the Ca^2+^-permeable AMPA (α-amino-3-hydroxy-5-methylisoxazole-4-propionate)-type receptors induces the death of spinal MNs in the rat in vivo and that this results in progressive paralysis. Acute or chronic infusion of AMPA in the lumbar spinal cord, by means of microdialysis [14,15] or osmotic minipumps [16], respectively, results in MN degeneration that correlated with motor alterations and finally paralysis of the rear limbs, in 3–12 h after microdialysis or in 3–5 days after the implantation of the osmotic pump. The acute MN death and ipsilateral paralysis caused by AMPA microdialysis perfusion was prevented or reduced by specific Ca^2+^-permeable AMPA receptor antagonists and by intracellular Ca^2+^ chelators [15,17], demonstrating the vulnerability of spinal MNs to the excitotoxic effect of Ca^2+^-permeable AMPA receptors activation in vivo, which had been previously reported only in studies in neuronal cultures in vitro [18-20]. Using the acute microdialysis procedure, which results in rapid ipsilateral MN loss and motor alterations of the corresponding rearlimb, we recently showed that among several energy substrates and antioxidants tested, pyruvate and β-hydroxybutyrate (βHB) notably protected against the excitotoxic effects of AMPA whereas the antioxidants tested were ineffective. These results allowed us to conclude that failure in mitochondrial energy metabolism was greatly involved in this rapid MN death, and also permitted to establish a correlation between the number of dead MNs and the degree of unilateral motor alterations [21]. Nonetheless, in this microdialysis acute experiment AMPA is perfused during 25 min and produces only unilateral paralysis, MN alterations and motor deficits that develop in less than 6 h, whereas the chronic slow continuous administration of AMPA through osmotic minipumps induces bilateral rear limb paralysis that progresses slowly along several days, also correlated with MN loss but extended to both ventral horns. Therefore, we considered of interest to evaluate the efficacy of the energy substrates and the antioxidants to protect against the chronic AMPA excitotoxic action. In addition, we correlated the protective effect along the progress of motor alterations with the MN loss in the infused region and in the immediate rostral region of the lumbar spinal cord, as well as with the prevention of astrogliosis induced by AMPA.

## Materials and methods

Adult male Wistar rats (280–300 g) were used in all experiments and were handled in accordance with international standards of animal welfare and with approval of the Institutional Committee for the Care and Use of Laboratory Animals (Approval No. RTI21-14). Rats were housed in a laboratory environment with a 12 h light/dark cycle and with food and water ad libitum. Animals showing complete paralysis and loss of the righting reflex within 15 s were sacrificed to avoid unnecessary suffering and were not analyzed further.

### Osmotic minipumps implantation

The implantation of the osmotic minipumps in the lumbar spinal cord was carried out essentially as previously described [16] with the following modifications.

All drugs were dissolved in 0.1 M phosphate buffer, pH 7.4. After some preliminary experiments with 7.5, 4.5, 3.5 and 1 mM AMPA (Tocris, Ellisville, MO, USA), the latter concentration was chosen because it induced a slow and progressive paralysis of the rear limbs along several days, whereas the effect of the higher doses was more rapid and severe. We tested the effect of the energy substrates pyruvate and βHB, and the antioxidants ascorbate and glutathione ethyl ester (GEE) (Sigma-Aldrich, Saint Louis, MO, USA) at concentration of 20 mM, based on our previous work with AMPA acute administration [21]. GEE was used instead of glutathione because it enters the cell more efficiently [22]. Osmotic minipumps (Alzet model 2004, capacity ~250 μL, flow rate 6 μl/day) were filled with one of the following solutions: vehicle as control, 1 mM AMPA, AMPA + sodium pyruvate, AMPA + DL-βHB sodium salt, AMPA + GEE, AMPA + L-ascorbic acid, and AMPA + antioxidant mixture (GEE + ascorbate), and they were incubated in sterile saline solution at 37°C for 48 h for stabilization before the implantation. The day of surgery rats were anesthetized with isoflurane 1-2% in a 95% O_2_ + 5% CO_2_ mixture and placed in a stereotaxic spinal unit. A longitudinal incision of the skin was made in the lumbar region and muscles surrounding the lumbar vertebrae were retracted. A stainless-steel screw (3.7 mm long, 1 mm diameter) was inserted on the left side of the third lumbar vertebra without reaching the surface of the spinal cord to anchor the implant. A 1–2 mm diameter hole was drilled at the same vertebrae and a small cut of the meninges was made to insert a borosilicate glass probe (1 mm long, 0.5 I.D. x 0.8 O.D., VitroCom Inc.) into the right dorsal horn of the spinal cord. The other end of the cannula was connected through a plastic tube to the osmotic minipump which was placed subcutaneously in the back of the rat, caudally to the skin incision. The probe and the screw were fixed to the bone with dental acrylic. At the end of the surgery, the skin incision was closed with surgical stainless-steel clips and rats received a single i.m. dose of penicillin (50 U). After recovering from anesthesia animals were kept in individual cages with water and food ad libitum until perfusion for histological analysis. Neither the control nor the experimental animals showed signs of pain or suffering during the time period studied, even during their performance in the motor tests.

### Assessment of motor function

Before surgery rats were trained to achieve 120 s on the rotarod test (Columbus Instruments), in which rotation started at 10 rpm and accelerated at 0.2 rpm/s, and also to perform a PGE task, where the latency to climb a vertical grid is scored. Three trials/day/rat were assessed in each motor test. In addition, the hind paw footprint was registered using non-toxic Chinese ink on the hind paws of rats. Besides, holding up the tail we photographically recorded the changes in the position of the rear limbs. These motor evaluations were assessed daily five days prior surgery until the seventh day, when animals were anesthetized and perfused for histology.

### Histology and immunohistochemistry

Seven days after osmotic pump implantation rats were perfused and fixed for histological and immunohistochemical analyses as previously described [16]. Briefly, spinal cords were removed, postfixed 48 h at 4°C and dehydrated with 20% and 30% sucrose solutions. Transverse sections (45 μm thick) of the spinal cords were obtained in a cryostat. Fifty serial slices at the site of the cannula infusion (infused region) and the next fifty rostral slices (rostral region) were obtained, so that the total tissue analyzed was 2.25 mm length, about 2 lumbar segments each region. Alternate slices were stained with cresyl violet or immunostained for neurofilament protein SMI-32 and glial fibrillary acidic protein (GFAP). The number of morphologically healthy MNs (i.e. large neurons with a soma diameter >20 μm and distinguishable nucleus, similar in appearance to those of the control and intact rats) in both the ipsilateral and contralateral ventral horns were counted in fifteen Nissl-stained slices/rat/region and the values were averaged.

For immunohistochemistry, mouse polyclonal anti-SMI-32 (1:500, Abcam), chicken anti-GFAP (1:1000, Abcam), as markers of neurons and astrocytes, respectively; Texas-red-conjugated anti-mouse (1:200, Invitrogen) and fluorescein-conjugated anti-chicken (1:200, Novex) antibodies were used. Sections were mounted on silane-covered slides and coverslipped with fluorescent mounting medium (DAKO). Sections were visualized under epifluorescent microscopy and merged images of the overlay of FITC and Texas Red channels were obtained with Image J (NIH, Bethesda, USA). The cross-reactivity in the immunofluorescent technique was excluded by control slices incubated in the absence of primary antibodies. There was no immunostaining in these controls.

### Statistics

The statistical analysis of the number of healthy MNs, the rotarod and PGE performance was carried out using ANOVA followed by a Tukey’s post hoc test. A value of p <0.05 was considered statistically significant.

## Results

### Energy substrates exert better protection than antioxidants against the AMPA-induced motor alterations

**Figure 1:**
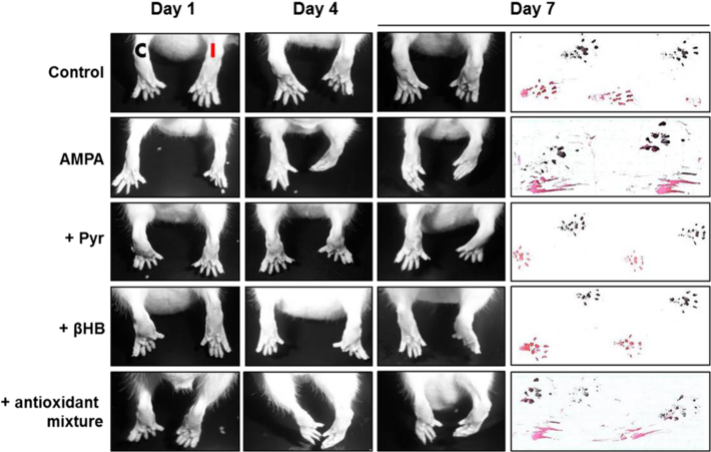
Progressive alterations of the rear limbs induced by AMPA and protection by energy substrates. Representative posterior views of the rear limbs (C, contralateral; I, ipsilateral) and hind paw footprints (red, ipsilateral; black, contralateral) at the initial (day 1), the middle (day 4) and last day (day 7) of treatment. The AMPA-induced progressive alterations were prevented by pyruvate and βHB but not by ascorbate and GEE alone (not shown) or in combination (antioxidant mixture).

**Figure 2:**
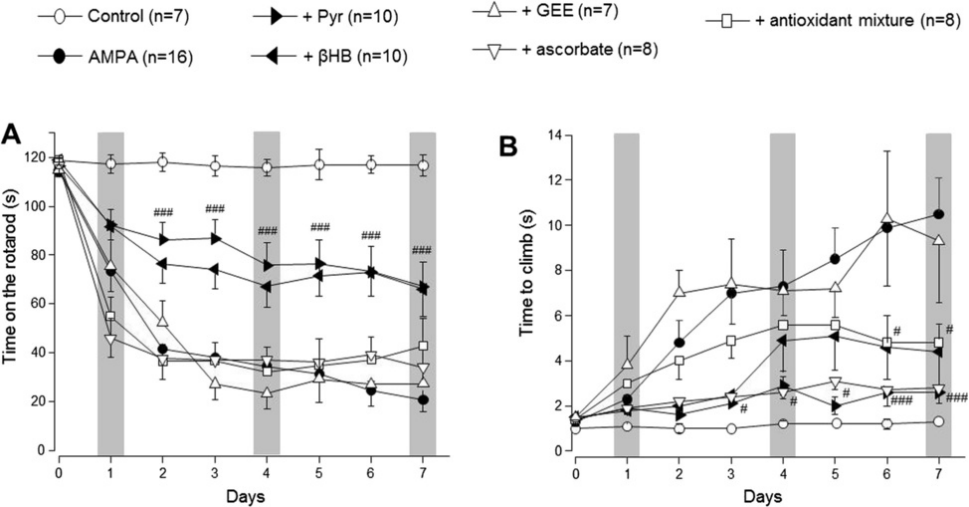
Assessment by rotarod **(A)** and PGE **(B)** performance of the protection by energy substrates. Pyruvate and βHB significantly prevented the notable motor deficits induced by AMPA in both motor tasks, whereas ascorbate improved the scores on the PGE test but not in the rotarod and GEE was ineffective. Three trials/day/rat were assessed in each motor task. Grey bars highlight the initial, middle and last day of infusion, as shown in Figure 1. Data are mean values ± SEM for the number of rats shown in parentheses. ^**#**^p < 0.05; ^**###**^p < 0.001 vs the corresponding value of the AMPA group.

**Figure 3:**
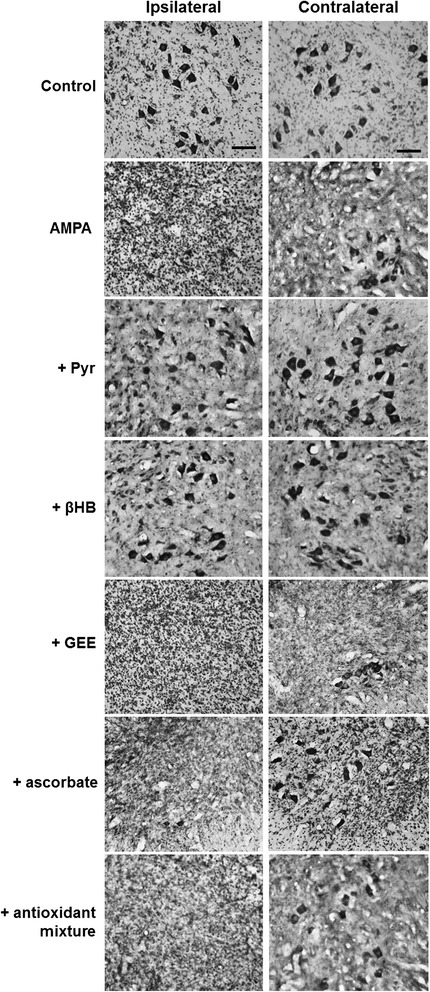
AMPA-induced MN degeneration and protection by energy substrates. Representative micrographs of the ventral horns of Nissl-stained sections of the infused lumbar spinal cord region of rats treated as indicated, seven days after minipump implantation. AMPA induced an almost complete loss of MN in the infused side (ipsilateral) and a less intense degeneration in the contralateral side, and both effects were clearly prevented by pyruvate and βHB but not by ascorbate or GEE. Quantitative analysis is shown in Figure 4. Scale bar = 100 μm.

**Figure 4:**
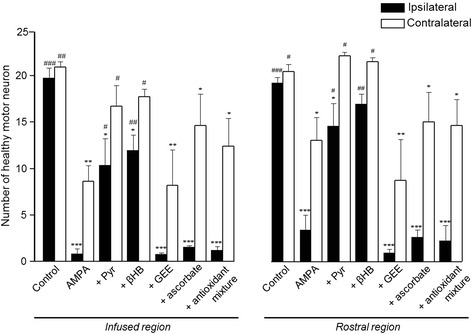
Quantitative analysis of MN loss in each experimental group. Number of healthy MNs in the ipsilateral and contralateral ventral horns in the infused spinal cord region and in the adjacent rostral region of rats treated as indicated, seven days after minipump implantation. Pyruvate and βHB notably prevented the AMPA-induced MN degeneration in the ipsilateral horn in both the infused and rostral regions, whereas ascorbate and GEE, alone or in combination (antioxidant mixture) were ineffective. Fifteen histological slices/rat/region were analyzed. Data are mean values ± SEM for the following number of rats in each group: control 7; AMPA 16; AMPA + Pyr 10; AMPA + βHB 10; AMPA + GEE 7; AMPA + ascorbate 8; AMPA + antioxidants mixture 8. *p < 0.05, **p < 0.01, ***p < 0.001 vs the corresponding side of the control group; ^**#**^p < 0.05, ^**##**^p < 0.01, ^**###**^p < 0.001 vs the corresponding side of the AMPA group.

**Figure 5:**
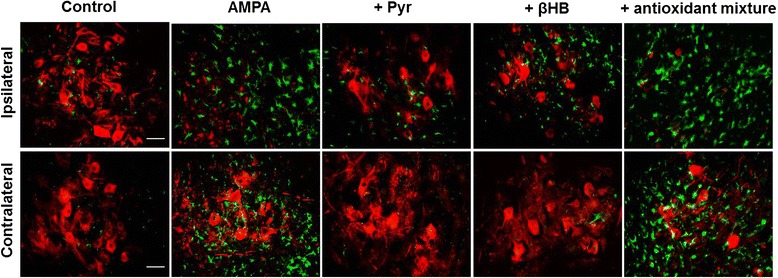
AMPA induced intense astrogliosis that was prevented by energy substrates. Representative immunohistochemistry of SMI-32 (red) and GFAP (green) of the ipsilateral and contralateral ventral horns at the infused spinal cord region of rats treated as indicated, seven days after minipump implantation. Note that in addition to the loss of MNs, AMPA induced a notable astrogliosis in both ipsilateral and contralateral ventral horns, and that this effect, as that on MN, was prevented by pyruvate and βHB but not by ascorbate and GEE, alone or combined (antioxidants mixture). Micrographs of the ascorbate or GEE alone groups (not shown) are very similar to those shown with the mixture. Fifteen slices/rat were analyzed (7 rats per group). Scale bar = 50 μm.

None of the control rats showed motor dysfunctions (Figures 1 and 2) or histological damage (Figures 3, 4 and 5) at any time up to 7 days, the maximum period studied. Perfusion of pyruvate, βHB, GEE or ascorbate alone at 20 mM concentration (n = 3 per group) was also innocuous (not shown). The chronic 7.5 mM AMPA infusion in the lumbar spinal cord of rats induced a gradual paralysis of the rear limbs in about 3 days [16]. As mentioned in Methods, in this work we reduced the concentration from 7.5 mM to 1 mM in order to extend the time of progression of symptoms. AMPA induced a paralysis that began in the distal ipsilateral rear limb (phalanges), ascending through the tarsus until the entire limb; the same process occurred with a short delay in the contralateral limb. This progression is shown in Figure 1 at days 1, 4 and 7. Quantitative assessment of these motor alterations in the rotarod and the paw grip endurance (PGE) test is shown in Figure 2, with emphasis in the values at these days to show the correlation with the position of the paws at these times. Whereas control rats remained in the rotarod for the total test period of 120 s, AMPA-infused animals fell at 73 s at day 1, 31–41 s at days 2–5 and 21 s at day 7. In the PGE task, control rats climbed always within 1 s whereas AMPA-treated animals at day 1 took double time to climb and then this time increased progressively until 7 s at day 4 and 10 s at day 7.

Perfusion of pyruvate and βHB partially prevented the AMPA-induced rear limbs paralysis. As shown in Figure 1, animals from these groups were able to partially control the ipsilateral phalanges and there was only a slight weakness in the contralateral ones, manifested by an incomplete tarsus footprint contralateral paw. Although the energy substrates did not entirely prevent the above described AMPA-induced paralysis, its protective effect was highly significant, attaining 91 s in the rotarod performance at day 1, and then 66–76 s from day 4 to 7 (Figure 2A). In the PGE task pyruvate was very efficient, since the time to climb was only 2–3 s during the whole observation period, whereas βHB was less effective from days 4–7, with climbing times of 4–5 s (Figure 2B).

In contrast to the notable protective effect of pyruvate and βHB, neither GEE or ascorbate, alone or in combination, prevented the AMPA-induced motor deficits observed in the rotarod. In these animals the times to fall were similar or even lesser (days 1 and 4) than those shown by the AMPA group during the whole period of observation (Figure 2A). In the PGE test (Figure 2B), GEE was also totally ineffective during all observation period, but ascorbate partially protected, especially during the first three days, where the time to climb was similar to those with pyruvate and βHB. However, when ascorbate was co-infused with GEE the protection during the first three days was diminished (Figure 2B), as shown also by the complete paralysis observed in the ipsilateral rear limb in these animals (Figure 1).

### Energy substrates reduced the AMPA-induced MN degeneration

As described in Methods, we analyzed two regions of the lumbar spinal cord (about two lumbar segments for each region). The infused region refers to the area where the cannula was implanted and the rostral region refers to the next rostral segments. We analyzed both regions because we found a differential effect of AMPA depending of its proximity to the probe. This pattern of damage correlates, as explained in the Discussion, with the progression of the bilateral paralysis.

As shown in Figures 3 and 4, the infusion of AMPA resulted at day 7 in an almost total loss of MNs (95%) in the ipsilateral horn and a reduction of 60% in the contralateral horn in the infused region, while in the rostral region the degeneration was also notable in both horns but less severe. The infusion of pyruvate and βHB significantly reduced the MNs loss in the infused region to 40%-50% in the ipsilateral horn and to only 10%-15% in the contralateral side. The protection by both substrates was also evident in the rostral region, where the number of healthy MNs was similar to the values in the control rats, especially in the contralateral horn. The infusion of the antioxidant GEE was ineffective to protect the spinal MNs in both regions, but ascorbate alone or in combination with GEE slightly reduced the number of MNs death, particularly in the contralateral horn of the infused region; these changes, however, were not significant (Figure 4).

Figure 5 shows that the loss of MN produced by AMPA in the infused region was accompanied by a remarkable astrogliosis, as detected by GFAP immunoreactivity, in both the ipsilateral and contralateral horns. Similarly to the protection of MN, both energy substrates tested almost completely prevented the glial reactivity in the contralateral horn and clearly reduced it in the ipsilateral side. In contrast, the GFAP immunoreactivity in the groups treated with AMPA + antioxidants alone or in combination was not different from the AMPA group. These results were reproduced in each of the 7 rats treated in each group of animals and quantitative analysis was not carried out.

## Discussion

In this work we show that the spinal MN death and astrogliosis induced by the continuous slow infusion of AMPA are prevented by pyruvate and βHB, and in one of the motor parameters studied by ascorbate, but not by GEE. Although the protection by the energy substrates was not complete, the number of protected MNs was enough to notably prevent the progression of the AMPA-induced motor deficits. These results show a clear correlation between the progress of motor alterations and significantly reduced MN loss and astrogliosis.

The AMPA-induced paralysis started in the ipsilateral phalanges and extended with time to the upper regions of the rear limb and to the contralateral side. This suggests that progression of the effects is due to a slow continuous diffusion of AMPA, explaining the larger damage in the infused region as compared with the rostral one. Because the infusion cannula was located between L3 and L4 segments of the spinal cord, the nearest MNs to the probe, that innervate the extensor digitorum that controls the phalanges movement are probably the first ones to receive AMPA and to degenerate, and then AMPA diffuses to affect MNs in the rostral region innervating upper limb muscles. The delay in the appearance of symptoms in the contralateral side is probably due also to the diffusion of the drug.

As mentioned in the Introduction, we have previously reported that pyruvate and βHB protected against the rapid deleterious action of the perfusion of AMPA during 25 min by reverse microdialysis, which induces unilateral paralysis and MN death in 3–12 h [21]. This acute effect allows just a restricted analysis of motor alterations and leaves open the question of whether these energy substrates are effective when the degeneration process is much slower, as it occurs in the osmotic pump continuous infusion procedure used in the present work. A therapeutic effect of oral administration of pyruvate was also reported in the mutant G93A-SOD ALS mice [23], although in these transgenic mice daily i.p. administration of pyruvate failed to delay the onset and motor decline [24]. Pyruvate or βHB also protected spinal MNs from mechanical damage [25], as well as striatal or hippocampal neurons from oxidative stress [26] or glutamate-induced excitotoxicity [27,28]. However, this is the first report of protection by pyruvate and βHB in a non-genetic model of spinal MN degeneration that correlated with the progression of motor impairment during several days.

It is noteworthy that there was no significant difference among the protective effect produced by pyruvate and βHB (tested at the same 20 mM concentration), thus suggesting that these substrates share a neuroprotective mechanism. A possible interaction of pyruvate and βHB with AMPA to explain the protection is improbable, because, judging from the structure of these molecules it is difficult to envisage the possibility of such interaction, especially considering that it should be occurring with both substrates and these molecules differ considerably. In addition, in our previous work using microdialysis [15,21], pyruvate and βHB perfusion started 60 min before AMPA and still protected. Furthermore, we have shown that pyruvate and βHB are also good protectors against glutamate-mediated excitotoxicity in the hippocampus, in an in vivo model that does not use AMPA but neurodegeneration is induced by stimulation of glutamate release by 4-aminopyridine [28]. On the other hand, these substrates share an important role in energy metabolism. It is widely known that pyruvate is the end product of glycolysis that can be oxidized by mitochondria driving the synthesis of ATP [29]**.** Pyruvate can also be taken by astrocytes and released as lactate to serve as energy substrate to neighboring neurons**.** βHB is a ketone body, a water-soluble mitochondrial fuel, that can be used by neurons when its concentration increases in the nervous system [30,31]. It is probably through these energy-linked mechanisms that pyruvate and βHB protect MN. In support of this interpretation, in cultured neurons these substrates protect against the excitotoxic damage produced by NMDA or glutamate, by stimulating the mitochondrial respiratory capacity and thus improving energy production [32-36], thus suggesting that in vivo they act through such mechanism. However, the protective effect of pyruvate and βHB has also been associated to the ability of α-ketoacids to neutralize H_2_O_2_ in a decarboxylating reaction. In cell cultures pyruvate protected neuroblastoma and striatal neurons exposed to H_2_O_2_ by diminishing the production of reactive oxidative species (ROS) [37,38], and βHB protected neuronal cultures exposed to kainate or glutamate also by reducing mitochondrial ROS production [33,34]. Improvement of the mitochondrial antioxidant defenses by MitoQ [39] or by the overexpression of antioxidant enzymes [40] also protected MNs from neurodegeneration induced by mutant G93A SOD1. Nevertheless, our results with ascorbate and GEE, an enzymatic cofactor and potent antioxidant, and one of the major antioxidant defenses of the cell, respectively, show that these compounds were ineffective, except for some improvement in the PGE task with ascorbate alone or in combination with GEE, which however cannot be correlated with a protection against MN loss. Thus, although a role of oxidative stress cannot be completely discarded, especially in the mutant SOD-linked MN degeneration, our results indicate that this mechanism is not the predominant factor in the MN death induced by chronic excitotoxicity, in agreement with our previous results with the acute infusion of AMPA [21].

An important factor in ALS appears to be a non-cell autonomous neuron death in which other cellular types, mainly glial cells, may play a role [41]. In this respect, our results show that AMPA induced a notable reactive astrocytosis that was, similarly to MN loss, prevented by the energy substrates but not by the antioxidants, suggesting that it is a consequence of MNs death rather than a direct effect of AMPA.

## Conclusion

This work shows that mitochondrial energy substrates protect spinal MNs from the neurotoxic cascade triggered by chronic excitotoxicity due to AMPA receptors overactivation, whereas antioxidants were not effective. We conclude that deficits in mitochondrial energy metabolism are an important factor in the mechanisms of this excitotoxic MN death in the spinal cord, while the role of oxidative stress is less significant. Because glutamate-mediated excitotoxicity has been postulated as a causative factor in MN degeneration in sporadic ALS, these findings suggest possible preventive or therapeutic strategies for the disease.

## Abbreviations

ALS: Amyotrophic lateral sclerosis
AMPA: α-amino-3-hydroxy-5-methyl-4-isoxazole propionate
GEE: Glutathione ethyl ester
GFAP: Glial fibrillary acidic protein
MN: Motor neuron
PGE: Paw grip endurance
ROS: Reactive oxidative species
SOD1: Superoxide dismutase 1
βHB: β-hydroxybutyrate
